# Development of a Culturally Appropriate Text Messaging Platform for Improving Breast Cancer Screening Uptake Among Ghanaian Women in Metropolitan Areas

**DOI:** 10.1155/2024/5587515

**Published:** 2024-10-24

**Authors:** Ransford Paul Selasi Sefenu, Adolphina Addoley Addo-Lartey, Harriet Affran Bonful, Adanna Nwameme, Timothy Agandah Abagre, Adolf Kofi Awua, Kofi Agyabeng, Kwabena Oteng Birimpong, Nii Armah Adu-Aryee, Florence Dedey, Richard Mawuena Kofi Adanu, Kolawole Stephen Okuyemi

**Affiliations:** ^1^Department of Epidemiology and Disease Control, University of Ghana, School of Public Health, Legon, Accra, Ghana; ^2^Department of Social and Behavioural Sciences, School of Public Health, University of Ghana, Legon, Accra, Ghana; ^3^Cellular and Clinical Research Center, Radiological and Medical Sciences Research Institute, Ghana Atomic Energy Commission, Kwabenya, Accra, Ghana; ^4^Department of Radiology, 37 Military Hospital, Accra, Ghana; ^5^Department of Surgery, University of Ghana Medical School, Korle-bu, Accra, Ghana; ^6^Department of Population, Family, and Reproductive Health, School of Public Health, University of Ghana, Legon, Accra, Ghana; ^7^Department of Family and Preventive Medicine, University of Utah, Salt Lake City, Utah, USA

**Keywords:** breast cancer, mammogram, mHealth, screening, SMS, text message

## Abstract

**Objective:** Early detection through screening could improve breast cancer (BC) outcomes in sub-Saharan Africa (SSA). We explored women's preferences for BC-related mobile health text messaging, described the development of a mobile-health text messaging platform, and examined the enablers and barriers to BC screening.

**Methods:** A concurrent mixed-method study of women aged 40–59 years was conducted. Four essential actions were carried out: (i) a baseline survey of 130 women, (ii) five focus group discussions (FGDs), (iii) a stakeholder meeting with BC research and clinical treatment specialists, and (iv) text message pretesting. The survey and FGD findings were used to create a culturally appropriate SMS platform for BC screening.

**Results:** Thirty-five text messages were developed and evaluated with the following communication goals in mind: 15 addressed BC awareness, six emphasized the importance of early detection, five alleviated anxieties as a barrier to BC screening, seven encouraged women to prioritize their health, and three indicated screening locations and costs. The majority (92.6%) of survey respondents who had heard of mammography (54/130) said screening was necessary. Fear of the screening procedure, receiving a positive diagnosis, and other testing-related worries (40.7%) were identified as potential barriers to BC screening, along with low income (18.5%), a lack of BC-related indicators (9.3%), insufficient breast awareness education (9.3%), and time restrictions (7.4%). The presence of BC-related symptoms (27.8%), breast awareness education (24.1%), and doctor's advice (16.7%) were all potential facilitators of BC screening uptake. The majority of FGD participants favored brief texts, with 42.3% preferring one text message per day.

**Conclusion:** Several factors limit women from accessing BC screening services; nevertheless, specific barriers such as a lack of BC education, time constraints, and disease fears can be successfully targeted through SMS messaging interventions to encourage women to use BC screening programs.

## 1. Introduction

### 1.1. Breast Cancer (BC) Morbidity and Mortality

BC is the most prevalent malignancy in women worldwide, with an estimated 2.3 million new cases in 2020, representing 11.7% of all cancers and almost one-fourth of all newly diagnosed cancers in women [1, 2]. The incidence of newly recorded cases and associated mortality are on the increase. These have been attributed to improvements in cancer registry systems and diagnostics [3], lifestyle changes [4], and the aging and growth of the population [1]. This incidence, however, varies by geographical location and age [5] and between transitioned and transitioning societies [1]. Despite making up only about half of the BC incidence worldwide, most BC deaths (62.0%) occur in low- or middle-income countries (LMICs) [6].

Factors such as late presentation with advanced disease [7, 8], ineffective treatment options, and poor health infrastructure [9] contribute to the low survival rates for women with BC in low- or middle-income county (LMIC) settings. In research on BC survival in Ghana, the 5-year long-term survival was 47.91% [10], as compared to a global population-based study on cancer survival, where the 5-year relative survival for BC was highest (> 80%) in North America, Sweden, Japan, Finland, and Australia, followed by Brazil and Slovakia (< 60%) and least (< 40%) in Algeria [11].

### 1.2. Early Detection of BC

Per current data, BC outcomes in LMICs are correlated with how early the malignancies are discovered [12]. The efficacy of early detection programs is driven by public education to stimulate active engagement in diagnosis and therapy, according to the Breast Health Global Initiative's recommendations for breast healthcare in LMICs [12]. Communication methods should also be adapted to the cultural environment, that is, the boundaries and taboos that accompany the diagnosis of BC, based on the social context and common healthcare belief systems [13].

The active engagement of women is one of the most important factors in the successful early detection of BC. Low educational and socioeconomic standing, limited knowledge about BC, poverty, unawareness, and poor comprehension of screening methods have all been cited as barriers to BC screening [14–17], as has the absence of physician referral [14]. Further research suggests that personal factors such as fear of the BC disease, potential mastectomy, low priority given to individuals' health, need for approval from close family members, fear of neglect and abandonment, and fear of dying from BC may all inhibit early detection [13, 18]. A positive family history of BC [18], doctor recommendations [19], and breast awareness in women who self-examine [20] appear to be variables that boost participation in BC screening.

In Ghanaian women, the greatest incidence of BC occurs in the 40–49-year age group, with a mean age of about 48 years at diagnosis [10]. The self-reported mammographic screening rate among Ghanaian women aged 40 years and older ranges from 2% to 3.6% [14, 21]. Presently, there is no formalized national program or policy for early diagnosis of BC, which may explain why most patients present with advanced stages of the disease, and about 52%–85% of patients often present with Stages III and IV disease [10, 22, 23]. In a study done at a tertiary hospital in Ghana, it was found that the stage of the disease at diagnosis correlated with mortality from BC, with more advanced stage disease having a lower survival disease [10]. Stages 0 and I had a 92% 5-year cumulative survival rate, while Stage IV had a 15% rate of disease [10].

### 1.3. BC Screening

Clinical breast examination is the cheapest and most readily accessible screening method. It is performed through palpation of the breast by trained health personnel. Although not very sensitive, it may be useful in detecting established BC in areas that lack other effective screening methods. It may follow breast self-examination (BSE) after a woman has noticed a lump in the breast, may be incidental during a clinic attendance for a non-BC-related issue, or is scheduled at a health facility as part of a screening protocol. The best method for detecting BC early is mammography screening [24]. This entails using X-rays to find breast abnormalities that are still not palpable. Mammography screening has been shown to reduce mortality rates globally by roughly 20% [24, 25]. However, lack of funding for mammographic screening programs in the majority of LMICs [24] can contribute to the late presentation of disease when treatment is less successful, more expensive, and less acceptable from an aesthetic perspective [10–12, 23].

### 1.4. Accessibility and Affordability of Mammography in Accra

In 2003, the National Health Insurance Scheme was established as a social intervention to replace out-of-pocket payments for healthcare in Ghana [26]. Currently, the NHIA covers standard treatment for cervical, breast, and some pediatric cancers. However, it does not cover BC screening and diagnostic procedures. In an urban area like Accra, mammography is available in both public and private institutions. The cost ranges from GHC200 ($16) in public institutions to GHC700 ($58) in private institutions. This is considerably expensive in a country where the minimum daily wage is reported as GHC18.15 ($1.52) in 2024 [27]. The high cost of mammography consequently places a financial burden on household incomes and impedes the receptivity of screening methods to eligible women. Our research was premised on the concept that the receipt of short message service (SMS) reminders with culturally acceptable educational content will help women justify the need to spend such sums of their household income on BC screening.

### 1.5. The Role of Technology in Health Compliance

Technology is already being applied in various forms in healthcare. Clinical Decision Support Systems (CDSS) are designed to augment clinical decision-making by providing patient-specific information to aid clinical and patient health-related decision-making [28]. This is associated with benefits such as reduced drug-drug interactions, misdiagnosis, and improved efficiency [29]. The use of mobile health (mHealth) technology such as SMS has been demonstrated to show improvement in the outcome of the management of chronic conditions such as Type 2 diabetes mellitus [30, 31]. SMS platforms are convenient because they are compatible with all types of phones and do not require internet connectivity.

### 1.6. SMS Reminders in BC Screening

Our approach to this study, which involves the use of SMS platforms to communicate health information, is informed by the fact that there has been an exponential increase in the use of mobile phone technology in Ghana over the last 20 years [32]. The implementation of mHealth technology has been associated with substantial improvements in health outcomes, notably in promoting behavioral changes that foster adoption and commitment to life-saving practices among the population [29, 33]. However, in developing countries, where most BC cases are diagnosed at advanced stages (III/IV), leading to poor treatment outcomes and high mortality rates [12], early detection through screening is crucial. To achieve this, there is an urgent need for vigorous health education to boost BC awareness and screening uptake, as has been underlined by studies on BC survival in Ghana [10]. Failing this, mortality due to BC in Ghana may worsen. To this end, we designed and pretested culturally appropriate SMS text messages that would influence Ghanaian women living in urban communities (40–59 years) to voluntarily screen for BC by mammograms. In doing so, we tapped into the availability of mHealth technology and applied mixed-method approaches to develop an SMS library. The text messages were created specifically to remove obstacles, strengthen enablers, and promote BC screening among Ghanaian women living in metropolitan areas.

This study describes the mixed-methods approach used to develop culturally tailored text messages aimed at increasing BC screening uptake among urban women in Accra, Ghana. It also reports the baseline knowledge and practices regarding BC among women enrolled in the baseline survey for a quasiexperimental trial that evaluated the effectiveness of the culturally tailored text messages in enhancing BC screening uptake.

## 2. Materials and Methods

### 2.1. Study Design and Participants

This paper is a methodological compilation of how mobile text messages were developed and pretested for use in a subsequent quasiexperimental trial to evaluate the efficacy of a culturally tailored program that promotes BC screening among women in Accra, Ghana. The focus of this article is to describe the processes leading to the creation of the SMS text message library and the ensuing pretest data and library. The paper further reports the baseline knowledge and practices regarding BC among women enrolled in the baseline survey for a quasiexperimental trial. This article was written per the principles specified in the consolidated criteria for reporting qualitative studies (COREQ) [34] (Supporting Information 1) and the Strengthening of the Reporting of Observational Studies in Epidemiology (STROBE) [35] (Supporting Information 2).

### 2.2. Study Sites and Sample Size

The baseline survey was conducted in the Greater Accra Region of Ghana, including the Tema and Accra sub-metropolitan areas, as well as the La Nkwantanang and Ashaiman Municipalities. Accra sub-metropolitan area and La Nkwantanang Municipality are in the western part of the Greater Accra Region, while Tema Metropolis and Ashaiman Municipality are situated roughly east of the region. These geographic divisions and the sites were purposefully chosen to reduce SMS intervention diffusion between the intervention sites (western) and control sites (eastern) during the subsequent primary intervention phase. The data collection tools and procedures were pretested among literate women working in the formal sector in the Shai Osudoku District in the Greater Accra Region.

The sample size for the baseline survey was estimated based on the following assumptions: (i) all women included in the study have never had a mammogram done, (ii) self-reported mammogram uptake was 2% among women in Accra [14], and (iii) sending mobile text messages resulted in a 15% higher absolute screening rate in participants who received the SMS intervention [36] and a 26% increase in the relative screening rate [37]. Assuming a 20% attrition rate and/or incomplete questionnaires, the estimated minimum sample size was 130 women.

**Figure 1:**
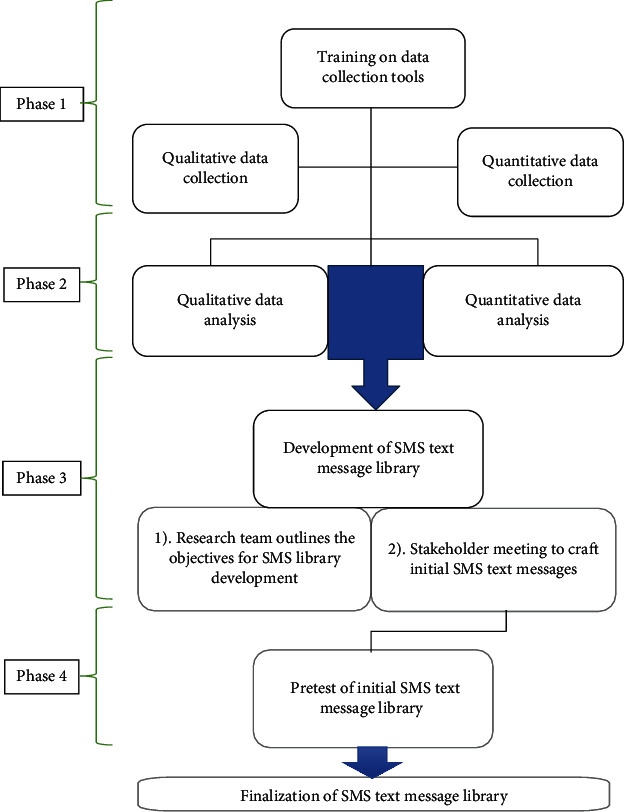
Flowchart of the study procedures.

Figure 1 depicts the study techniques used in the development of a culturally appropriate text messaging platform to increase BC screening uptake among Ghanaian women in metropolitan locations. The primary outcome of the study was the creation of SMS text messages to encourage BC screening. The study employed a concurrent mixed-procedures strategy in which both quantitative and qualitative methods were carried out simultaneously. A formative evaluation was undertaken to collect demographic information, health history, understanding, and perceptions of BC screening through focus group talks and a quantitative survey. The data was then utilized to develop a mobile text message library in collaboration with stakeholder engagements, which was then pretested with a small, randomly selected group of women from four districts in Ghana's Greater Accra Region.

The study population comprised women who had never had a mammogram. Participants in the formative phase (both focus group discussions [FGDs] and surveys) were simply required to be between the ages of 40 and 59; literacy was optional. In the pretest stage, women had to be between the ages of 40 and 59, have access to at least one personal phone, and pass a screening literacy exam. The current research methodologies are comparable to those employed by Bonful et al. [38] during the development of a culturally adapted SMS intervention to increase cervical cancer screening uptake among Ghanaian women in urban areas.

### 2.3. Phase 1: Training on Data Collection Tools, Concurrent Qualitative, and Quantitative Data Collection

#### 2.3.1. Training on Data Collection Tools

The field team was trained for 2 days on data-collecting instruments, sample methodologies, and consenting protocols at the University of Ghana's School of Public Health. They also received training on how to use the Open Data Kit (ODK) tool for electronic data collection. All the data collection methods for the quantitative survey, as well as the interview guide for the FDGs, were subsequently pretested over 2 days in Dodowa, Shai Osudoku District, in the northern portion of the Greater Accra Region, with 14 women. Using simple balloting, two eligible women were chosen randomly from a bank, while the remaining participants came from Dodowa communities. Following the initial pretest, the study team convened to discuss the electronic questionnaire and interview guide. In total, the study team held four separate sessions to finalize all data gathering techniques.

#### 2.3.2. Qualitative Data Collection

FGDs were held between February and May 2017, utilizing pretested interview guides to gather information about participants' views and understanding of BC screening and mammography testing. Five FGDs with 5–8 women per session. Discussions were held among women aged 40–59, recruited from banks, and municipal assembly workers in the La Nkwantanang Municipality. Nonprobability purposive sampling was utilized to choose participants, and only women who met all the inclusion criteria and were willing to participate in the study were included. FGDs lasted about 50–90 min. The project coordinator and fascinator was one of the study coinvestigators who had prior experience collecting qualitative data and was also a final-year PhD student in the School of Public Health, Department of Epidemiology and Disease Control. She moderated each focus group conversation while the field research assistants took notes.

All FGDs were held in a private conference room at the University of Ghana's School of Public Health or La Nkwantanang Municipal Assembly and audio-recorded with the participant's consent.

#### 2.3.3. Quantitative Data Collection

We sought potential participants from both formally recognized institutions and within the communities. The formally recognized institutions were primarily financial institutions, with a few representatives drawn from district assemblies and university campuses. The initial target audience for our study was older women (40–59 years old), but we were unable to achieve the needed sample size for the survey by relying solely on bank recruiting. As a result, we had to modify our protocol to include women at other formal institutions, which required further ethical approval. The modification was necessary because many women working at banks were often younger than we had anticipated (under 40 years old). Bank employees are a community of urban working women whose health-seeking behaviors are severely limited by their work schedules; hence, finding time for screening activities would require significant motivation. We reasoned that if sending targeted SMS messages might motivate these women to have breast exams and/or mammography screenings, the same method would most likely work for other groups of urban working women. Furthermore, we recruited women from varied socioeconomic backgrounds to assess how well our SMS intervention works in different populations. Because bank employers have specific standards for recruiting banking professionals, we anticipated that women working in all areas of the bank would hold a tertiary degree. When compared to people with lesser levels of education, those with higher levels of education earn more money; hence, participants working in banks were expected to have relatively higher incomes than women in the communities. Banks also had regular business hours, which allowed us to schedule interviews based on the preferences of both the woman and her employer.

The research team acquired data on the overall number of banking institutions in the study sites by driving around all the key streets in the study locations and generating a list of all the banks and financial institutions located on either side of those main routes. All female employees present at work were informed about the study, and those who were eligible and willing to participate had a literacy exam before recruitment. To recruit participants from the community, a residential household within 5 km of the nearest participating formal institution (bank, district assembly office, or tertiary institution) was randomly chosen as a starting point using the blindfold and point technique. Subsequent research participants were recruited using a systematic selection procedure, which involved selecting every third residential house along the community's principal access roads. One eligible woman was chosen at random from each selected residential home and invited to participate in the study. When a home had more than one eligible woman who wanted to participate in the study, a simple ballot was utilized to choose one.

Between February and May 2017, data were collected utilizing a structured questionnaire on a Samsung Galaxy (Table 1) (SMT231) running ODK software using face-to-face interviews. All interviews lasted about 45 min and were conducted in English, both in banks and communities. The gathered data included women's demographic and baseline variables such as age, marital status, education, employment, income, health status, family history of cancer, health insurance, and intention to obtain a mammogram. We also gathered data on previous and current smoking history, knowledge of BC screening, and screening barriers and facilitators.

### 2.4. Phase 2: Data Management and Analysis

#### 2.4.1. Qualitative Data

For the qualitative data, the verbatim transcriptions of the notes and recordings generated during FGDs were entered into MAX QDA 2018 for data management and analysis. After the research team examined the data, a coding framework was created and applied to segments of the transcripts. The team identified initial themes from the coded segments, and these led to the construction of subthemes and global themes (Table 2), following the steps outlined by Guetterman, Fetters, and Creswell [39].

#### 2.4.2. Quantitative Data

Quantitative data was exported from ODK in comma-separated variable (CSV) format and imported into STATA 14.0 (Stata Corporation, College Station, Texas) for management and analysis. Categorical data were described using frequencies, while the summary of the age of respondents (continuous) was reported as median with interquartile range because it was skewed. Chi-square tests of associations or Fisher's exact tests of independence were used to compare the responses of women recruited from formal institutions with those from the communities. All statistical tests of the hypothesis were conducted at a 5% significance level.

### 2.5. Phase 3: Development of Text Message Library

#### 2.5.1. Objectives for SMS Library Development

The following objectives were established by the research team to direct the creation of the text message library based on the findings from the baseline cross-sectional survey and the FDGs: (i). to provide knowledge about BC and the significance of early detection; (ii) to assuage the fear of the disease and encourage screening and treatment; (iii) to encourage potential participants to make time for their health in general and breast health in particular; and (iv) to provide information on access, cost, and availability of BC screening.

#### 2.5.2. Type of Information on BC to Be Included in the SMS Messages

Respondents to the quantitative survey proposed a wide range of SMS message topics, the majority of which focused on BC awareness (Supporting Information 3). They recommended education on BC, its causes and risk factors, how to prevent it, how to treat it, the need for mammography screening tests, the cost of a mammogram, and where to get one.

#### 2.5.3. Stakeholder Meeting to Develop Initial Text Messages

With these goals in mind, a stakeholders' gathering was organized in June 2017 at the University of Ghana's School of Public Health to develop the initial text messages and decide on their frequency and delivery hours. The stakeholder meeting was attended by the research team and experienced BC and clinical care researchers from the Ghana Health Service, Ministry of Health, and Ghana Atomic Energy Commission. Stakeholders who could not attend the meeting submitted comments in advance based on a summary report for the baseline survey and formative qualitative study that was shared a priori. The meeting covered presentations on the scope of the work, a summary of the baseline survey results, and FDGs highlighting the key facilitators and barriers to BC screening uptake, what content participants expected to see in the SMS library, and the mode of message delivery. The stakeholders' discussion resulted in the creation of well-phrased, short text messages organized around the five communication objectives.

#### 2.5.4. Creation of the Evaluation Tool (the Text Message Library)

The initial text messages produced during stakeholder discussions are shown in Supporting Information 4. The messages were grouped based on the communication goals which were informed by the FGD and baseline survey findings. These goals included BC awareness, highlighting the importance of early detection, allaying anxiety as a barrier to BC screening, encouraging women to prioritize their health, and helping women identify locations for screening and the associated costs.

Following the first part of the meeting, a range of three to nine messages were selected for each communication goal to create an evaluation tool of 35 messages (Supporting Information 5), which would later be used to assess the effectiveness of the text message program in promoting BC screening in a quasiexperimental trial. Of the 35 text messages developed during the first part of the stakeholder engagement, 15 text messages addressed BC knowledge, six emphasized early detection, five addressed fears as a barrier to screening, seven encouraged women to prioritize their health, and three provided cost and screening location information. During the second part of the stakeholders meeting on the same day, individuals who were unable to participate in person were sent the first round of text messages for feedback before the final text message library was created.

### 2.6. Phase 4: Pretesting of the Evaluation Tool

The evaluation tool of 35 text messages was pretested for 2 weeks in July 2017. The pretest was administered to 10 randomly selected women from formal institutions in East and West Legon, as well as the Madina Municipality. We took care to ensure that these women had not participated in the previous focus groups or the baseline survey. At the commencement of the pretest study, the messages were coded and loaded onto the platform of Hubtel Ghana Limited, a provider of SMSs. The women received SMS messages from Hubtel Ghana Limited twice a day, at 9 a.m. and 2 p.m. The delivery dates and timings for each message were determined. Following the pretest, the performance of the test message library and delivery was assessed using a modified assessment tool previously used in a study to assess the efficacy of text messages for outpatient malaria case management in Ghana's Greater Accra Region [40].

Each of the 35 text messages transmitted was assessed to ensure that the women received, read, and understood each message. The length and wordiness of the text messages were also considered, as were any expressions that made the messages confusing or difficult to comprehend. Respondents who requested to rewrite any of the text messages received were allowed to do so. The respondents' perspectives on the applicability of the messages in increasing BC screening in Ghana were carefully documented. In addition, the pretest participants' general concerns about the delivery structure were examined. These included how long it took respondents to read the texts and when they typically read them (e.g., during work hours, breaks, or after business hours), their experience with the program, the aspects of it that they found most and least interesting, whether there were any parts that they found confusing or irritating, and the appropriateness of the text volume.

The pretest also assessed whether the evaluation tool influenced respondents' attitudes toward BC screening, the acceptability of the SMS promotional messages, and whether they wanted future SMS text reminders about BC screening from the National Noncommunicable Disease Control Program sent to their phones. Phone access during business hours as well as mobile network coverage issues were reported. The research team subsequently analyzed the pretest data and finalized the culturally appropriate text message library and delivery mechanism for use in the subsequent quasiexperimental trial (findings not reported here).

### 2.7. Ethical Considerations

The Noguchi Memorial Institute Medical Research provided ethical approval (certified protocol number [CPN]: 011/16-17). Before recruiting any available individuals, the district chief executives of all research locations, as well as the heads of departments/managers of banks and all other formal organizations involved in the study, were contacted for authorization. All eligible participants were taken through an informed consent process in which all study methods were thoroughly discussed. Only those who stated that they understood the study procedures and signed the informed consent paperwork were included in the study. To protect participants' anonymity and confidentiality, women were assigned arbitrary numbers, and these numbers were used to identify women during focus groups. Only the researcher knew the participants' identities. During FGDs, surveys, and pretests, participants were encouraged to express themselves openly and without fear of criticism. Participants could leave or stop answering questions if they felt uncomfortable. All procedures were carried out per the guidelines in the Helsinki Declaration of ethical principles for research involving human subjects.

## 3. Results

### 3.1. Sociodemographic Characteristics of Participants—Phase 2

A total of 130 women were recruited for the baseline survey, 69 from the community and 61 from formal institutions. The median age of the participants was 46.0 years (IQR: 42–52). Most women (93.9%) identified as Christians and 66.2% of them were married. Almost half of the women (47.7%) worked as public or civil servants. All the recruited women had some level of formal education because it was an inclusion criterion.  Table 3 summarizes the respondents' sociodemographic features.

### 3.2. Sociodemographic Characteristics of FDG Participants

Thirty-one (31) women aged 40–67 participated in the FDGs. Five FGDs were held with participants of various employment statuses (Table 4).

### 3.3. Knowledge of BC, Causes, Risk Factors, and Prevention—Phase 2

#### 3.3.1. Awareness of BC

From the survey data (Table 1), nearly all the women (97.7%) had heard of BC disease. All women at formal institutions had heard of BC as a disease, while only three women in the community had never heard of it. Discussions in the focus groups also indicated that most of the women had heard of BC and knew that it typically manifests as a lump or growth in a woman's breast. Many of the participants also recounted stories about family members and neighbors who had battled the sickness and subsequently died. The following quotes underscore these insights: I think it's a lump that comes into the breast and that's the source of the cancer. (p3, 62y/o, trader, FGD 2)

For breast cancer, we used to hear about it, but we didn't know what it was like until last year when my mother suffered from it. When it started, it was like a boil, her breast was swelling so we thought it was just a normal boil. It was later when she went to the hospital that we got to know it was breast cancer. (p3, 42y/o, trader, FGD 1)

I have also witnessed breast cancer before. One of our neighbors from Achimota was a victim of breast cancer –one of her breasts was bigger than the other. Her own [disease] started from the nipple, and because those days there was no treatment for breast cancer, she lived with it till she died. (p1, 44y/o, trader, FGD 1)

What I know is that one of my cousins had it. The lump started coming. It was too late when she got to know it was cancer so by the time she got to the hospital they had to cut the breast. Gradually when they were treating her, eventually she gave up (died).… Then the same thing happened to the senior one too. So that's how I got to know about breast cancer. (p2, 47y/o, unemployed, FGD 2)

#### 3.3.2. Knowledge About the Risk Factors of BC

Of the 127 women in the survey who reported ever hearing of BC, approximately one-third (34.6%) knew the disease's cause or risk factors. Compared to community respondents, more women at formal institutions were aware of the causes of BC (43.2% vs. 56.8%). Some of the suggested causes of BC in the FGDs include smoking, drinking, poor nutrition, high cholesterol, and obesity. Other risk factors for BC, reported by respondents, include consuming fatty and junk meals, reusing vegetable oils, eating products in cans that are nearing the end of their shelf life, and having early menarche. The following narratives support these assertions:

Cigarette causes cancer, for example, if you are sitting close to someone and the person is smoking, you might end up getting cancer because of the smoke you inhale…if you're a woman you can get breast cancer and if you're a man you can get throat cancer. If a woman inhales the smoke every day, it will accumulate in her breast, and she can get breast cancer. (p1, 44y/o, trader, FGD 1)

Sometimes the diet, our diet, the food we eat, especially when we are around 40 years and above- fatty foods, junk foods, and those kinds we should be careful or move away from it. And then I also heard some people say if you menstruate very early you have the chance of getting breast cancer. (p2, 44y/o civil servant, FGD 4)

Most of the canned food they normally reduce their prices and say they are doing promotion, but most are expiring products, and we buy them because we feel they are cheap, but they can cause cancer…Yes, taking alcoholic drinks too can cause cancer. (p1, 44y/o, trader, FGD 1)

Respondents reported typical beliefs and misconceptions regarding the risk factors of BC, such as wearing second-hand (preowned) or tight brassieres and utilizing brassieres as pockets for phones or money. Others believed that eating hot food packaged in polyethylene bags, preparing food with microwaves and heaters, applying cosmetics, and indulging in sexual activity that could result in a bite to the breast all increased a woman's risk of developing BC. The following accounts support these assertions:

What I heard is that some of the women who put money in their brassiere can get breast cancer. I also think that when you put a phone in your brassiere pocket, you can get breast cancer. (p5, 50y/o, civil servant, FGD 4)

Some people wear [brassieres] too much. They wear it all day when they are at home. They even sleep with it. It can cause [breast] cancer. (p1, 45y/o, hairdresser, FGD 3)

I have also heard that when you buy this second-hand brassiere and you don't wash it well, it can also cause it [breast cancer]. (p6, 49y/o, civil servant, FGD 5)

I also heard that when you continue to eat food from the microwave, it possible you can also get it [breast cancer]. (p5, 50y/o, civil servant, FGD 4)

A friend of mine once said, when you are having sex with your husband or boyfriend you should not allow him to suck your breast because he can bite your breast and through that, you can get breast cancer. When they bite your breast, there is this minor pain you might not pay attention to, but the pain will increase with time and might lead to breast cancer. (p1, 44y/o, trader, FGD 1)

#### 3.3.3. Knowledge About the Prevention of BC

Among the 127 women in the survey who had heard about BC (Table 1), more than half (58.3%) knew how to prevent it, and this knowledge was higher among women enrolled in formal institutions (60.8%). During the FGDs, many participants saw routine medical check-ups as an important step in BC identification. They added that because BC can run in families, everyone in the family should get regular checkups. Furthermore, it was underlined that having regular check-ups would aid in early diagnosis and, as a result, early treatment of the disease. Other recommended precautions include decreasing weight, limiting dairy products, and self-examination of the breasts. The following quotes demonstrate these points:

It is necessary for an entire family to go for the screening if they get to know a member of the family has it. This type of sickness is like a family property- once it affects one person the rest are likely to get it too, especially if you eat and drink from the same bowl. (p1, 44y/o, trader, FGD 1)

It is only when we go for a check-up regularly that we will be able to know whether we have it or not, then we can start treating it early because the earlier the better. (p5, 41y/o, trader, FGD 1)

The self-examination of the breast should be so frequent that is every month when we are getting to menopause. So, the young ones, when they are about to menstruate can still do the self-examination and then we the elderly ones too I think every 2 to 3 months we should try to do your self-examination so if you see anything that you are not comfortable with, you report on time. (p2, 44y/o, civil servant, FGD 4)

Although we are not aware of any licensed herbal therapies for preventing BC, our focus group participants believed that several herbal medicines and traditional practices could help prevent the disease. Again, eradicating traditional practices targeted at stopping the breasts from budding was also seen as a preventive measure for BC. Furthermore, some of the participants stressed the need for men to show less enthusiasm for women's breasts while relating with their partners. The quotes below resonate with these views:

It is because of this breast cancer that is why people don't joke with this “noni juice” and the rest; they use that to prevent themselves from getting the cancer. (p1, 44y/o, trader, FGD 1)

Traditionally, when a child is growing up and starts to have the breast coming out, some use cloth and wooden sticks to bind and interrupt the formation of the breast. These things should be avoided. (p3, 42y/o, civil servant, FGD 4)

Having the adult men playing too much with your breasts, they should avoid that as it can cause breast cancer. The breast belongs to the infants. It is no more the preserve of the adult men. (p3,52 y/o, civil servant, FGD 5)

Some of the women admitted to not knowing what steps to take to prevent BC, and they advocated for widespread education to enlighten women about the causes, methods of prevention, and available therapies. As a result of this lack of information, women who felt cancer was caused by supernatural forces sought treatment outside of conventional approaches.

We don't know how it comes so we don't know how it can be prevented. Or what brings the breast cancer. If I know what brings fever, I will know how to protect myself but we don't know what brings breast cancer, so we don't know how we are going to protect ourselves. (p1, 59y/o, housewife, FGD 2)

…I think the most important thing is education. People do not even know, that is why if people get this disease, instead of going to the hospital or seeking medical attention, they divert to other places because they associate it to a lot of supernatural beliefs. (p3, 42y/o, civil servant, FGD 4)

On the other hand, some of the FGD participants who discussed BC treatment were ignorant of any available options, while others could mention treatment modalities such as surgery (lumpectomy and mastectomy) as well as chemotherapy. The women also stated that a doctor's early diagnosis of the illness was critical for a successful course of treatment. Some women suggested herbal remedies, prayers and deity consultations, urine treatments, and other ways to address the condition. The quotes below demonstrate these opinions:

There's this doctor, Aunty Grace, she is in Kumasi. She talks about chemotherapy- I think that is the only treatment I have ever heard. (p3, 52y/o, civil servant, FGD 5)

If it [breast cancer] is detected early, they can remove the lump without it spreading, so the early detection is very important. (p2, 44y/o, civil servant, FGD 4)

Yes, they go for chemotherapy; they can also cut off your breast. This is to prevent the disease from spreading and affecting the other breast. (p3, 42y/o, trader, FGD 1)

Herbal treatment can be used to cure breast cancer, though it's a bit questionable because you need to be certain about what you are taking in. Some also say the cure is through divine intervention by powerful men praying for you. Some also think when you go to the deities- they also have a way of taking the disease away. (p3, 52y/o, civil servant, FGD 5)

I heard someone too using early morning urine to wash down every day. (p7, 49y/o, civil servant, FGD 4)

### 3.4. Awareness of Screening for BC

Table 1 presents information on BC screening awareness. Less than half (41.5%) of the women recruited for the study had heard of mammography tests as a BC screening approach. The number of participants who had ever heard of mammography testing was similar for women recruited from the community (42.0%) and formal institutions (41.0%). The majority (92.6%) of those who had heard of the mammography test believed it was necessary to get one, with 74.1% intending to take the exam and 18.5% doubtful. More women from formal institutions desired to have a mammography screening than women recruited from the community (88.0% vs. 62.1%). Of the 54 women who had heard of the mammography test, the majority (61.1%) knew of at least one site where they could get the test done; nevertheless, the majority (96.3%) were uninformed of the cost.

Throughout the FGDs, it was clear that the participants understood the basic notion of screening for various illnesses such as diabetes, HIV, and malaria. Some respondents claimed they were aware of breast examinations and mammograms for BC screening, and a few said they had had one. The stories below highlight these elements:

The nurses taught us how to do it, you lie down on your back and use your hands to massage your breast to find out if there is a lump in…. They said that your husband can do it for you if you know you cannot do it yourself. (p3, 42y/o, trader, FGD 1)

I have done a mammogram before even though that was not the reason why I went to the hospital that day. I went with abdominal pains and the doctor ended up screening me after he got to know I had not been screened for breast cancer before. (p2, 40y/o, trader, FGD 1)

Although most respondents believed BC screening was beneficial, they also observed that many women did not participate in the program at health centers due to the related costs. They advocated offering free or reduced-cost screening services, as well as public awareness efforts. The following quotes express these sentiments.

Not necessarily free but the charges should be something small. Let's say the total cost for the treatment is GhC500.00 (approximately USD 50) and the government subsidizes it to GhC200.00, everybody will go…. They have been announcing it, but we have not been able to go because we do not have money. (p5, 56y/o, trader, FGD 1)

If it is free and there is much education, that one at least you can get 8 out of 10 women going for the screening. (p1,40 y/o, civil servant, FGD 5)

### 3.5. Source of Information on BC Screening

**Figure 2:**
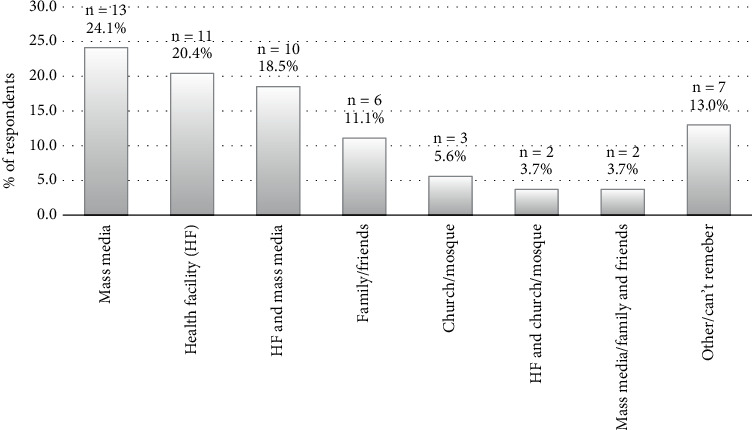
Source of information on breast cancer screening among Ghanaian women in selected urban communities.

Most (63.0%) of the information about BC screening with a mammogram came from the health facility and the mass media. Information from friends and family came next (11.1%). The other sources of information were the internet, radio, and places of worship (Figure 2).

### 3.6. Barriers to BC Screening Among Respondents

**Figure 3:**
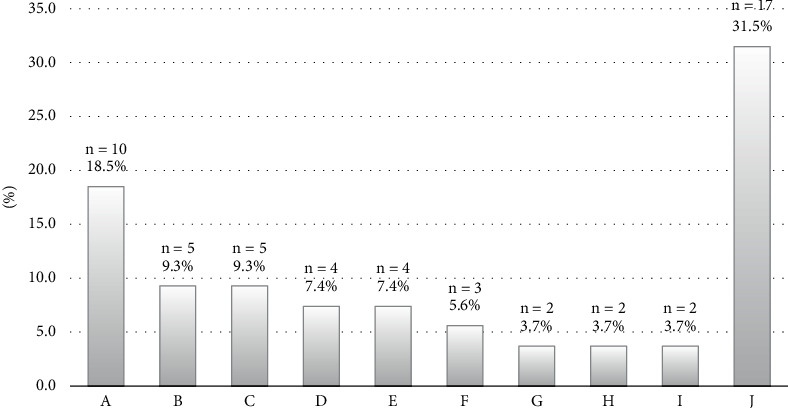
Barriers to breast cancer screening among Ghanaian women in selected urban communities. (A) Low-income level. (B) Lack of signs and symptoms. (C) Low education on breast awareness. (D) Lack of time. (E) Low-income level and lack of signs and symptoms/lack of time/fear of breast cancer/fear of breast cancer and neglect. (F) Fear of screening procedure/death. (G) Lack of service provider. (H) Fear of breast cancer. (I) Lack of service provider and lack of time/lack of time and fear of breast cancer. (J) Other fears/family history.

The factors that prevent BC screening varied across the 130 survey participants. Some of these barriers included the fear of being diagnosed with BC, the cost of the mammography test, the absence of breast-related disease, a lack of time to schedule the test, a lack of BC awareness education, and anxiety about the screening process.  Figure 3 shows the hurdles to BC screening in our population.

Similar findings from the FDGs were observed. The women mentioned a range of reasons for not obtaining BC screenings. Some of these included a lack of financing, concern about an impending diagnosis or surgery, hours-long lines at test centers, and the distance to tertiary medical facilities where the tests are typically performed. The following FGD narratives describe these feelings:

At times too that fear that you may have it, you wouldn't like to go… You have the feeling that I may have it, I will go, and it may be detected and they may choose to cut my breast off, so I won't go. (p6, 55y/o, civil servant, FGD 4)

Last week, I was feeling pains in my breast, but I was scared to go and screen because of that thing they put your breast inside [the mammography machine]. I learned they will use the machine to press your breast. The way people described the process [it] scares me. Money was also a factor; I prefer to use herbal medicine because I do not have enough money for the hospital treatment. (p1, 44y/o, trader, FGD 1)

Time is another factor that will prevent people from going. People will not go if they will have to pay after wasting their time in a long queue but if it is free then it will compensate for the time they spent there waiting for their turn to the screen. (p2, 40y/o, trader, FGD 1)

We have Madina Polyclinic, Kekele Polyclinic, and Pentecost Hospital all in Madina. If you want us to go to Korle-bu or Ridge or Tema, that will be too far for us. (p1, 59y/o, housewife, FGD 2)

Other reasons provided by respondents for not accessing BC screening services were cultural or religious constraints, the belief that the disease has a spiritual component, illiteracy, and self-medication. Furthermore, several women believed they did not require a screening if they were healthy and had no symptoms.

There should be more education because I know some women are not supposed to expose themselves to any other person apart from their husbands [due to religious beliefs]. So, such a person wouldn't like to go [for breast screening]. (p2, 41y/o, civil servant, FGD 5)

But I'm not sick so I don't see why I should waste my time and go [for breast screening]. (p1, 59y/o, housewife, FGD 2)

Some people think it is a spiritual thing so they don't believe they should take it to the hospital. (p6, 49y/o, civil servant, FGD 5)

Some of them because they have not been to school, even if they have it, they wouldn't know, and they will not go and check to see what is there [in the breast]. Some of them will just go to the pharmacy and buy any drug because of the pain so before they will get to the hospital, things have gone worse. (p2, 47y/o, unemployed, FGD 2)

Barriers at the healthcare facility level were also identified throughout the conversations. The respondents recalled moments when they felt shy and nervous because there were so many staff members in the examination room. Access to services was also cited as a barrier due to some nurses' poor patient care and some health workers' casual manners. Furthermore, positive screening results raised issues regarding confidentiality, as the women feared stigma if their diagnosis became public.

And the thing too is that these nurses, when they are doing it [the test], you will see about three or four people in the room and they ask you to remove your dress. I feel bad when it happens like that. It happened to me, and I nearly stopped the test. This woman [a nurse] picked up iron scissors and used them to pick cotton balls, I looked at it and I said, what is going on? It scared me from the beginning. (p5, y/o, civil servant, FGD 4)

Sometimes when you go to the medical centers, the nurses are very snobbish, which would put you off. You would want to persevere to do it later, after all, I am not sick now, then you go back when you feel sick. (p3, 52y/o, civil servant, FGD 5)

I will prefer to go to a private hospital, where I will be attended to quickly and leave, rather than being in the queue and the ones who are going to do the test will be distracted with talking on their cell phones when people are in the queue. (p2, 41y/o, civil servant, FGD 5)

Most people do not want to go and check because they are afraid somebody may leak out the results and they will be pointed at as having breast cancer. (p3, 52y/o, civil servant, FGD 5)

### 3.7. Facilitators of BC Screening Among Respondents

**Figure 4:**
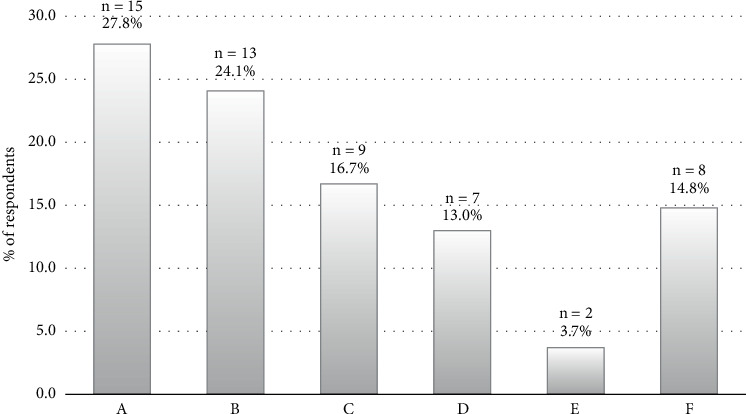
Facilitators of breast cancer screening among Ghanaian women in selected urban communities. (A) Showing signs and symptoms of breast cancer. (B) Breast awareness education. (C) Doctors' recommendation. (D) To know my status. (E) Doctors' recommendation and high-income level. (F) Other reasons including family history; doctors' recommendation, high-income level and showing signs and symptoms; doctors' recommendations and to know their status; high-income level and showing signs and symptoms of breast cancer; and nearness to a service provider.

Women who participated in the study went for a mammogram for several reasons, including demonstrating signs and symptoms of the disease, receiving BC awareness education, a doctor's recommendation, and a desire to learn their status. Other facilitators include being aware of a family history of BC, having a high income, and living close to a screening facility, among others.  Figure 4 summarizes the reasons mentioned by respondents for making BC screening easier.

Despite the numerous barriers to BC screening indicated by FGD participants, the women identified some factors that may motivate women to use the service. These thoughts supported what was found in the survey. Most of the women stated that, while most women would only get screened if they began to develop BC symptoms, having test centers close to their homes would encourage them to use the service right away. In addition, it was suggested that the government provide free or subsidized screenings to encourage women to participate. The following quotes elaborate on these ideas:

Others will only go if you detect symptoms of cancer, for that one you won't even wait for someone to come and call you before you go… If you check yourself and you feel there is a lump or pains in your breast, you would like to go and check what is wrong with you. (p2, 40y/o, trader, FGD 1)

Proximity is important. People would want to go for screening, but they may not have the money to transport themselves to the venue. But if you were to site it at Madina market, women from around will come because it is much closer. (p3, 52y/o, civil servant, FGD 5)

If the government can support by subsidizing the cost of the treatment for us. I am sure everybody including myself will go for the screening test. (p5, 56y/o, trader, FGD 1)

Aside from the fear, the treatment is very expensive, so support from the government will help. I think the fear will reduce if you know at the end of the day the government will support you financially for the treatment. (p1, 44y/o, trader, FGD 1)

### 3.8. Practices of Women Regarding Receiving Health Information by SMS Messages

In the quantitative study, almost all the women surveyed reported having ever received an SMS on their mobile phones (99.2%), while 57.5% had received health-related messages. The majority of the surveyed participants (97.7%) stated they normally read the SMS messages (Table 5).

All the FGD participants had received health-related messages from friends, family, doctors, or network providers. However, the great majority of texts were shared via WhatsApp. As seen by the accounts presented below, some of the women also admitted that they had ended their subscriptions to these services because they were too expensive or bothersome.

Recently I received one, but it was through WhatsApp. These days people prefer using WhatsApp. (p3, 42y/o, trader, FGD 1)

I received one from my first cousin entreating me to try and go for a breast cancer test and to forward to colleagues on the same platform. (p3, 52y/o, civil servant, FGD 5)

They deduct money from your account [for health messages so people don't like it]. They will tell you a day is 75p or so and it's not everybody who can afford it, so people ignore and tell them to stop. (p1, 45y/o, hairdresser, FGD 3)

When the health-related text is plenty then I will put the phone off. I will not read it. (p1, 57y/o, housewife, FGD 2)

### 3.9. Willingness to Receive SMS Messages on BC

#### 3.9.1. Positive Response to BC Screening SMS Messages

In the quantitative survey, the majority (96.9%) of the women interviewed said they would be willing to receive SMS messages promoting them to attend BC screenings (Table 5). In addition, most of the women (90.0%) reported that receiving SMS messages would influence their willingness to go for BC screening. When asked what features of a text message urging BC screening would elicit a positive response, the FGD participants agreed that the message should make it apparent that the screening will be provided for free or at a reduced cost. The women indicated that to inspire women to act, the language should also highlight the impact of BC on women's health, nearby screening places, and general BC information. However, the message should not be alarming because it will deter women from accessing screening facilities rather than encourage them to do so. These opinions are expressed in the quotes below.

Yes, you should let them know the amount involved is less and you should state the amount. The message should also include specific locations for the screening test, and the process shouldn't be too long: it should be something simple. (p2, 40y/o, trader, FGD 1)

The message should not include something that will put fear in people. You should also tell them there is a treatment for the disease if their diagnosis turns out to be positive. The screening can be free but if the people are not sure there is a treatment for the disease they will not come for the screening. Most people have this perception that if you get cancer that is the end of your life, so it's up to you to convince them otherwise. (p1, 44y/o, trader, FGD 1)

Before you start screening, you will definitely be giving them tips on the disease for some time, like how dangerous the disease is and how it can kill. (p1, 45y/o, hairdresser, FGD 3)

People would like to hear that early detection will save your life. (p8, 50y/o, civil servant, FGD 4)

The women also proposed sending a notice the day before the screening, using simple language and enticing slogans, as well as offering refreshments at the BC screening venue. The following are extracts that support these claims.

I don't have a problem if you send me a message ten times to remind me once it's about health. The first message should come with the program, that is, the date, time, and everything, so the subsequent ones can serve as a reminder. But don't forget to send one a day before the program and it should read “Tomorrow the program is coming on live. (p1, 44y/o, trader, FGD 1)

Please use simple words that we can read and understand. When we get the message, we will also send it to our friends. (p5, 56y/o, trader, FGD 1)

Please the refreshment too is important as soon as it is mentioned that after the screening, we will give refreshments, people will come in their numbers. (p4, 44y/o, civil servant, FGD 4)

#### 3.9.2. Negative Response to BC Screening SMS Messages

A few women (3.1%), all of whom were from formal institutions, indicated displeasure with receiving health-related SMS messaging (Table 5). Similarly, some participants in the FGDs predicted unfavorable responses to text messages if receivers were overwhelmed by the volume of texts they were getting or if too much information was offered at first contact. Some respondents suggested that some women may assume the testing places will give inadequate services, discouraging them from undergoing the exam. The following stories highlight these perspectives:

But if we say everything in the text message, the person has been briefed with everything she needs to know so she will not see the need to go for the test. A little about the disease, not everything. (p2, 40y/o, self-employed, FGD 3)

You have to sit down to select the important messages which will push the women to go for the screening but if you tell them everything that we said in this discussion, the information will be too much. (p1, 59y/o, housewife, FGD 2)

…Sometimes when you go to the medical centers, the nurses are very snobbish, they would put you off. So, you can't find your bearings around the way to the screening, but you would want to persevere to do it. But after all, I am not sick then [when I came] and then you must go back with your sickness…. (p3, 52y/o, civil servant, FGD 5)

Being in the queue and the ones [nurses] who are going to do the test will go round picking calls [personal phone calls] while people are in the queue…I would prefer to go to a private hospital, where I will have whatever, I am going to do done quickly and leave, rather than these free, free. (p2, 41y/o, civil servant, FGD 5)

### 3.10. Preference for Receiving SMS Messages During the Day

#### 3.10.1. Preferred Frequency and Duration of SMS Messages

In terms of the preferred frequency of BC SMS messages per day, 42.3% of survey participants preferred receiving one message per day, 16.9% preferred receiving two, 6.6% preferred receiving three, and 5% preferred receiving more than three messages per day. Approximately 29.2% offered no suggestions. About half of the survey respondents (50.8%) were unaware of the optimal length of an intervention period for receiving BC SMS messaging. However, 21.5% of respondents indicated that the intervention should last more than 2 months. The remaining suggested timeframes are less than 2 months. These sentiments were consistent among individuals from formal institutions and the community (Table 5). The discussants in the FGDs also shared their thoughts on the frequency of message distribution. Some women stated that regular messaging was required to ensure that the women received the knowledge. Other women, however, thought that receiving constant text messages would be annoying. Some women preferred receiving SMS three times a week, while others preferred receiving them once or twice a week. It was also suggested to send out a notice just before a screening program began.

It's not annoying. Some people would like to read it because they know they are reading to educate themselves and some will ask, “Why are these people sending me these text messages?”, so they will not read it. (p2, 40y/o, self-employed, FGD 3)

Three times a week because as for me, I stopped using the Airtel-Tigo network because of these messages every day. So, I think three times a week will be alright. When the message is coming too much you will get bored. (p6, 49y/o, civil servant, FGD 5)

…Getting it every day, sometimes you may not read it. But if twice a week, it will be ok. (p1, 45y/o, hairdresser, FGD 3)

So, if you send one today then another one in a week to remind us of, and another one when it is getting closer to the screening time then you remind us again. (p1, 44y/o, trader, FGD 1)

#### 3.10.2. Preferred SMS Message Volume and Length

The FGD participants were asked how lengthy they wanted the text messages to be to ensure that they were read by the recipients. Though a few of them stated that they would read the messages regardless of length, the majority suggested a few lines of roughly three phrases. They also suggested making the messaging memorable and to the point. The narratives that follow emphasize these points.

It should be short and then strong and meaningful. (p2, y/o, civil servant, FGD 4)

As for me, you see my phone- if the message is plenty, how can I read it so if I get 5 or 4 lines it's ok. (p3, 52y/o, trader, FGD 2)

You should make it catchier and have everything in there with substance because me when you give me plenty things I won't read. (p3, 52y/o, civil servant, FGD 5)

It shouldn't be too much. If they see a message like that some of them will not even look at it, they will ignore it because it's too long. (p2, 40y/o, self-employed, FGD 3)

I like reading, so I don't mind if the messages are plenty. (p1, 57y/o, housewife, FGD 2)

#### 3.10.3. Preferred Timing of SMS Messaging

Most survey respondents (54.6%) stated that they would not mind receiving SMS messages at any time of day; however, some (17.7%) preferred receiving SMS messages in the evening (Table 5). When compared to their community counterparts, fewer women in formal institutions favored receiving SMS messages at any time of day (65.2% vs. 42.6%). Many of the women who took part in the FGDs agreed that the text messages should be sent in the afternoons when most women take their workplace meals. They said that they were too busy with domestic responsibilities in the mornings and nights. Some women, however, preferred receiving the messages in the morning so that they could read them before going about their day. To ensure that recipients make time in their calendars for BC screening. It was also advised that women receive reminder messages encouraging them to get checked. Some of these recommendations are summarized as follows:

In the morning everybody is rushing out and in the evening for women, we come rushing to cook for our families; and after cooking we are tired, and we just lie down and sleep. But in between times, let's say in between the lunch times, from noon up to about 2 pm, most women are not that busy and engaged depending on what they do. If you are in the office, that time frame is your lunch time so if you have a very strict boss, you have your lunch that is when you can just steal a glance at your phone. (p3, 52y/o, civil servant, FGD 5)

It should be in the afternoon because most of the workers go for lunch in the afternoon during lunchtime or after work. (p2, 40y/o, self-employed, FGD 3)

Me morning, early morning between 6 to 7, I go through my WhatsApp, my messages before I come to the office. (p6, 49y/o, civil servant, FGD 5)

If you send the message today, then it means am going for the screening tomorrow but if you send the message today and you want me to be there today, my schedule may not permit me. (p1, 59y/o, housewife, FGD 2)

### 3.11. Acceptance of BC Screening SMS Messages

Many participants in the FGDs believed that the concept of text messaging would be acceptable to women who receive it, particularly if they were literate. Some believed that using text messaging to educate women about BC was an important step toward preventing the disease. Some respondents, however, were skeptical that text messaging would be helpful, believing that illiteracy would be a barrier. This was an understandable opinion given that the FGDs were done during the formative period, so the participants could be illiterate or literate. To be included in the text message library pretest, women had to be literate. Finally, several women claimed that refusing to seek medical assistance when healthy would hinder recipients from responding to the messages. The following quotes support these views:

The text messages can serve as a preventive measure for breast cancer. It will create awareness. (p6, 55y/o, civil servant, FGD 4)

Sending text messages will work. If am here and I have seen a message about free screening for breast cancer, I would like to go and know if I have the disease or not. (p3, 40y/o, trader, FGD 3)

Me for instance, I can't read, so when I get a text message it will just be on the phone till my husband deletes them…he will tell me the message is not important. (p1, 44y/o, trader, FGD 1)

If I don't feel any pain in my breast and you sent me a text message, I will not go. (p4, 59y/o, trader, FGD 2)

Many respondents believed that providing information just via text message was insufficient. To guarantee that illiterate women were not disadvantaged, they recommended that community education or house-to-house education be provided in addition to SMS messaging. Some women also proposed using the WhatsApp messaging network, which was deemed to be more accessible and less expensive than SMS texting, as well as visual aids such as television and photographs to supplement the information in the texts.

Checking the messages means reading them, what about us who cannot read but want to benefit from this information? It is also embarrassing to send the message to someone to read for me, unless someone very close to you, someone you can share your secret with. For example, let's say we pick a thousand women and it's only three or five hundred that can read, what happens to the rest who cannot read? So please you can send the text messages and still do the door-to-door education for the sake of those who cannot read. (p1, 44y/o, trader, FGD 1)

Now we have WhatsApp groups so you can send the information there. I think that one will be more convenient and less expensive than the SMS where you have to send it to all the contacts one by one. But no problem if you think you can afford to send the SMS. (p3, 42y/o, trader, FGD 1)

You can also put it on the television. On the phone they will not see what the disease is like. (p3, 40y/o, trader, FGD 3)

I think the messages must at times be in pictures. When the thing is in picture form, we like viewing it. (p6, 49y/o, civil servant, FGD 5)

### 3.12. Findings From the Pretesting of SMS Messages—Phase 4

A pretest exercise was carried out prior to the main intervention deployment. This required sending 35 SMS messages to 10 randomly selected women aged 18–39 years to solicit feedback on the content and delivery times of the texts. Each participant received two text messages per day for 2 weeks. Only three of 10 women who planned to participate in the pretest study did so, yielding a 30% response rate (Table 6). All three pretest respondents claimed to have received and read all 35 text messages sent to their phones. Furthermore, all three participants believed that the 35 messages were clear and of appropriate length or intensity.

When asked when they read the text messages, two of the three participants said they did so during the workday, while the third said she did so frequently after the office closed. Only two of the three pretest participants found the timing of the text messages appropriate. When questioned about the importance of SMS messages in encouraging BC screening uptake, all three pretest respondents agreed that the SMS message's content could encourage them to undergo the screening. Furthermore, every pretest responder expressed a desire to receive SMS reminders from the National Noncommunicable Disease Control Program for BC screening.

## 4. Discussion

In this formative qualitative investigation and baseline cross-sectional study, we describe the methods for creating a culturally relevant tool to boost BC screening uptake among a small sample of middle-aged women in Ghana's metropolitan city of Accra. We also examine women's preferences for general health-related SMS text messages and BC screening messaging.

### 4.1. Barriers to BC Screening

Recent research suggests that a variety of factors, including women's lack of knowledge and awareness of the illness, may hinder them from using BC screening services [41]. Participants in the current study identified a wide range of screening difficulties. Notably, 44% of our survey respondents cited low income, the absence of illness-related symptoms, limited awareness of BC, and a lack of time as the most common barriers to BC screening. Women in the FGDs had similar worries about the cost of the screening, the worry connected with a diagnosis or imminent operation, the time lost at the test sites owing to long queues, and the distance traveled to the healthcare facilities to be screened. Our findings are consistent with previous research in Asia [42] and the sub-Saharan African region [43–46]. A survey of female community pharmacists in Jordan found that the absence of BC symptoms was the most common reason for not performing a BSE or clinical breast exam [42]. Similarly, community studies in Kenya [44] and Tanzania [46] found that a lack of BC-related signs and symptoms is a barrier to breast healthcare. In line with the findings of our current study, a Ugandan study identified distance to screening sites and poverty as important structural barriers to BC screening [45], while a Nigerian study identified a lack of BC awareness, cost, and perceived need as the primary reasons for not having mammograms [43]. Other challenges to BC screening have been reported elsewhere. For instance, a systematic review of 17 studies identified discomfort and embarrassment associated with screening procedures, anxiety about the results, a lack of health insurance, the belief that screening is unnecessary, and a lack of professional guidance as major barriers to mammography screening among minority women in the United States [43]. Many of the barriers to BC screening highlighted in our study can be overcome by raising public awareness of the benefits of early detection. To reduce the cost of screening for women from low-income families, BC screening could be added to the list of services covered by National Health Insurance Plans.

### 4.2. Facilitators of BC Screening

The following criteria were major motivators for our research participants to undertake BC screening: a doctor's suggestion, BC awareness education, the presence of BC symptoms, and a personal interest in finding out one's BC status. These observations were corroborated by emerging themes from our qualitative data, in which FGD members identified proximity to testing facilities and government screening cost refunds as possible BC screening enablers. Our findings are consistent with those of other similar studies conducted in Ghana [14, 47–49] and elsewhere [50–52]. Opoku, Benwell, and Yarney's study of Ghanaian women [14] and other similar studies conducted worldwide [18, 53–55] indicate that if a doctor recommends it, women may be enthusiastic to undertake BC screening. This outcome is not surprising given that women are more likely than men to seek diagnosis and treatment because they believe their doctor can manage the disease [56]. In many cultures, medical practitioners, including doctors, are regarded as “trusted confidants,” making them a useful resource for persuading women to get BC screenings. In many cultures, medical practitioners, including doctors, are regarded as “trusted confidants,” making them an effective resource for persuading women to undergo BC screening. Furthermore, public health initiatives in the media can be a useful way to raise awareness and provide information about BC. For example, a community-based intervention program that included a short and widespread media campaign to promote BC awareness and screening resulted in a higher reduction in Korean women's perceptions of BC-related myths [57]. Similarly, a cross-sectional survey of women in Southeast Nigeria indicated that a media campaign resulted in an increased level of BC awareness [58], and in the United Kingdom, a mass media campaign boosted awareness of the connection between alcohol and BC [59]. Thus, health education, particularly when delivered by health professionals through the media, can facilitate cancer screening among women [56].

We found that the most prevalent reason for women to be examined for BC is the presence of symptoms and indicators of the disease. Our findings are comparable with those of Abu-Helalah et al. in Jordanian women, who identified the presence of signs and symptoms associated with BC as a possible facilitator of screening [52]. A cross-sectional survey conducted in rural Ghana observed that women who had participated in a program on the benefits of early BC detection using the BSE technique were much more likely to perform BSE regularly [49]. Research among asymptomatic Ethiopian women who had never had BSE found that this was mostly due to a lack of knowledge of BC screening approaches [51], indicating that BC awareness education could be a primary motivator for screening uptake. Another research among asymptomatic Jordanian women found that almost half (49.3%) of those who had never had a mammogram agreed or strongly agreed that they should wait until a lump or other signs of BC occurred before being examined [52]. These findings lend weight to the idea that BC education can induce asymptomatic women to participate in early screening. Women in our study also stated that having test sites close to their homes would encourage them to use the service immediately. This perception is supported by the findings of a scoping investigation of facilitators and barriers to cervical and BC screening in SSA, which identified geographical access as the most likely factor in facilitating early identification of BC [56].

### 4.3. Women's Preferences for SMS Texting in General, Especially Those About BC

Text messages transmitted via mobile devices, in addition to BC awareness campaigns, can be utilized to address knowledge gaps and BC screening service acceptability. Text messaging has been used successfully to encourage behavior change and improve healthcare service delivery [60–62]. Understanding women's willingness to receive text messages, as well as their preferences for the content, frequency, duration, and length of the text messages, can aid in the development of a text message delivery program that increases the likelihood that health-related text messages are read by the intended audience. In this study, almost all survey respondents (96.9%) indicated a willingness to receive BC-related SMS text messages. The FGD participants had similar sentiments, urging that the SMS messages include information on the cost of BC diagnostic testing, such as whether the treatments will be free or subsidized. We are unaware of any previous studies in SSA that investigated women's willingness to receive text messages for BC screening. However, research undertaken in other regions has found a wide range of behaviors in women's willingness to accept SMS messages concerning BC prevention. For instance, 36% of rural women in New Mexico [63], 46.1% of university students in Malaysia [64], and 51.2% of female college students at a southwestern American university [65] reported a desire to receive BC prevention SMS messages. Our data show that women are particularly interested in receiving SMS messages about BC prevention. This could be because most of our participants could read and understand written material. This could be because most of our participants could read and comprehend written material, which may have a bearing on their health-seeking behavior. Kratzke, Amatya, and Vilchis support the idea by reporting that lower levels of self-efficacy are closely associated with higher levels of education among American college women [65]. Given that text message intervention programs have been shown to raise cancer screening rates [66–68], the findings of this study can be used to establish policies that promote BC screening in the SSA region.

Although almost all study respondents said they would be willing to receive text messages for BC screening, their opinions on the optimum number of SMS per day differed. Furthermore, participants' preferences for the duration of a text message-delivered BC prevention program vary slightly. Our FGD participants' opinions were likewise diverse, with some believing that sending out text messages regularly would encourage people to attend a BC screening session, while others believed that doing so would irritate potential program participants. These findings agree with the study results of Singleton et al. [69], who observed that participants' preferences for text message frequency, duration, amount, and timeliness can differ significantly. It will be critical to understand whether these variations are random or due to participant characteristics. This is because these changes, if not random, indicate that a targeted messaging approach may be more effective in promoting BC screening in women.

The timing and duration of SMS messages for health intervention programs also vary across studies. In a narrative review paper of 162 studies in which daily or weekly SMS reminders of varying lengths were provided to participants, researchers determined that the reminders increased patient compliance and served as appointment reminders in nearly every trial [70]. In one mixed-methods study, four messages were sent weekly to enhance the emotional and physical health of cancer survivors. According to the authors, most survey participants thought the number of messages they received each week was normal; nevertheless, many focus group members said it was inappropriate to receive texts “every day” [66]. Another qualitative study was aimed at developing a text message intervention program to prevent cervical cancer in Korean American immigrants who received three SMS each day for a week to a month [68]. Participants in those FGDs believed that the number of text messages sent and the duration of the intervention were enough. When asked about the ideal length of texts, our FGD participants preferred short texts, which is consistent with Chernick et al. [71] and Lee et al. [72], who found that American adolescent girls and Korean American immigrant women preferred short text messages for health promotion. Another study on mHealth text message preferences found that a similar proportion of students preferred both long and short texts [73]. Again, understanding the link between these variances and participant characteristics is crucial to facilitating BC screening uptake.

### 4.4. Pretest of Text Message Library

Our text-messaging program pretest results indicate that when designing a health-related text message intervention program, it may be beneficial to provide a range of text message delivery schedules so that potential program participants can select the times they prefer to receive messages. Another approach is to conduct a pilot poll to learn about prospective program users' preferred text message delivery times. Some studies [72, 74, 75] employed pretest investigations to guide the construction of health-related text message intervention programs. Pretesting SMS text messages before further implementation may provide alternative perspectives on the required content and test message distribution strategy. In Lee et al.'s research to develop a mHealth intervention to promote Papanicolaou testing and human papillomavirus immunization in an underprivileged immigrant population, focus group participants preferred appealing messages [72]. The study also requested feedback from focus group participants, who were subsequently sent 15–20 messages per day for 7 days, depending on the topic of the day. Similarly, the authors changed the texting curriculum from 18 to 22 SMS text messages and the delivery period from 14 to 16 days in response to feedback from eight pretest participants in a study to develop a spiritually-based text messaging program to raise cervical cancer awareness among African American women [74]. In contrast, Somera, Mendez, and Mummert's study revealed that while pretesting an SMS to increase cervical cancer screening uptake among Chuukese women in Guam was successful, it failed when SMS frequency was gradually increased [75].

### 4.5. Study Limitations

Although the present study's findings support the usage of mHealth technology in promoting BC screening, there are some limitations to how they can be applied. To begin, the generalizability of our findings to all Ghanaian women may be limited because the women in this study were literate and lived in cities. Because our baseline investigation was formative, we are unable to evaluate the potential effects of confounding on the variables analyzed. Future studies can use more robust designs that allow for a comprehensive evaluation of confounding variables. Nonetheless, the findings of this study may serve as a catalyst for future research into the factors that impede and encourage BC screening in women of SSA background. Furthermore, a text message intervention to improve BC screening could potentially be culturally tailored to take advantage of women's text messaging preferences.

## 5. Conclusions

The study's findings provide an essential understanding of the key barriers and enablers to BC screening uptake, as well as women's preferences for the content and structure of SMS messages. Our findings highlight the need for opportunistic BC screening efforts and to educate asymptomatic women about BC screening options and facilities. Engaging physicians in targeted BC counseling for at-risk women at contact points could be a promising approach to boost BC screening in at-risk groups.

## Figures and Tables

**Table 1:** Breast cancer and breast cancer screening awareness: knowledge, attitudes, and practices.

**Characteristics**	**Community**	**Formal institutions**	**Total**	**p** ** value**
	**n** ** (%)**	**n** ** (%)**	**n** ** (%)**	
Heard of breast cancer disease				0.099
Yes	66 (95.7)	61 (100.0)	127 (97.70)	
No	3 (4.4)	0 (0.0)	3 (2.30)	
Knows of causes/risk factors				0.149
Yes	19 (28.8)	25 (41.0)	44 (34.60)	
No	47 (71.2)	36 (59.0)	83 (65.40)	
Knows of breast cancer prevention				0.001
Yes	29 (43.9)	45 (73.8)	74 (58.30)	
No	37 (56.1)	16 (26.2)	53 (41.70)	
Heard of the mammogram test				0.904
Yes	29 (42.0)	25 (41.0)	54 (41.50)	
No	40 (58.0)	36 (59.0)	76 (58.50)	
Necessary to go for a mammogram test				0.408
Yes	26 (89.7)	24 (96.0)	50 (92.60)	
No	1 (3.4)	1 (4.0)	2 (3.70)	
Do not know	2 (6.9)	0 (0.0)	2 (3.70)	
Intend to do a mammogram				
Yes	18 (62.1)	22 (88.0)	40 (74.10)	
No	2 (6.9)	2 (8.0)	4 (7.40)	
Undecided	9 (31.0)	1 (4.0)	10 (18.50)	
Knows the place to do a mammogram test				0.037
Yes	14 (48.3)	19 (76.0)	33 (61.10)	
No	15 (51.7)	6 (24.0)	21 (38.90)	
Knows the cost of mammogram test				0.915
Yes	1 (3.5)	1 (4.0)	2 (3.70)	
No	28 (96.6)	24 (96.0)	52 (96.30)	

*Note:* %: column percentage.

**Table 2:** Themes and subthemes emerging from FGDs among Ghanaian women in selected urban communities.

**Global themes**	**Subthemes**
Knowledge of breast cancer	Awareness of breast cancer Knowledge about risk factors for breast cancer Knowledge about the prevention of breast cancer

Awareness of screening for breast cancer	Source of information on breast cancer screening Barriers to breast cancer screening Facilitators of breast cancer screening

Receipt of health information by SMS messaging	General health information by SMS messaging Willingness to receive SMS on breast cancer Preference for receiving SMS messages on breast cancer screening Acceptance of SMS messaging for breast cancer screening

**Table 3:** Sociodemographic profile of Ghanaian women in selected urban communities.

**Background characteristics**	**Community (** **N** = 69**)**	**Formal institutions (** **N** = 61**)**	**Total (** **N** = 130**)**	**p** ** value**
	**n** ** (%)**	**n** ** (%)**	**n** ** (%)**	
Median age (LQ, UQ)	46 (42, 52)	46 (42, 52)	46 (42, 52)	
Ethnicity				0.017
Akan	31 (44.9)	26 (42.6)	57 (43.9)	
Ga/Dangme	23 (33.3)	9 (14.8)	32 (24.6)	
Ewe	8 (11.6)	19 (31.2)	27 (20.8)	
Other (specify)	7 (10.1)	7 (11.5)	14 (10.8)	
Religion				0.857
Christian	65 (94.2)	57 (93.4)	122 (93.9)	
Islam	4 (5.8)	4 (6.6)	8 (6.2)	
Traditional	0 (0.0)	0 (0.0)	0 (0.0)	
None	0 (0.0)	0 (0.0)	0 (0.0)	
Other (specify)	0 (0.0)	0 (0.0)	0 (0.0)	
Marital status				0.396
Married	41 (59.4)	45 (73.8)	86 (66.2)	
Cohabiting	1 (1.5)	0 (0.0)	1 (0.8)	
Divorced	4 (5.8)	5 (8.2)	9 (6.9)	
Separated	7 (10.1)	4 (6.6)	11 (8.5)	
Single	9 (13.0)	4 (6.6)	13 (10.0)	
Widowed	7 (10.1)	3 (4.9)	10 (7.7)	
Level of education				< 0.0001
Primary/middle/JHS	22 (31.9)	10 (16.4)	32 (24.6)	
SHS/O-level/A-level	28 (40.6)	14 (23.0)	42 (32.3)	
Vocational/technical	13 (18.8)	2 (3.3)	15 (11.5)	
Tertiary	6 (8.7)	27 (44.3)	33 (25.4)	
Other (specify)	0 (0.0)	8 (13.1)	8 (6.2)	
Occupation				< 0.0001
Public/civil servant	8 (47.7)	54 (88.5)	62 (47.7)	
Trading	34 (49.3)	0 (0.0)	34 (26.2)	
Artisan	9 (13.0)	0 (0.0)	9 (6.9)	
Unemployed	7 (10.1)	0 (0.0)	7 (5.4)	
Student	0 (0.0)	7 (11.5)	7 (5.4)	
Retired	3 (4.4)	0 (0.0)	3 (2.3)	
Other (specify)	8 (11.6)	0 (0.0)	8 (6.2)	
Access to NHIS:				0.077
No NHIS card	27 (39.1)	15 (24.6)	42 (32.3)	
Have NHIS card	42 (60.9)	46 (75.4)	88 (67.7)	

*Note:* %: column percentage.

Abbreviations: JHS, junior high school; LQ, lower quartile; NHIS, National Health Insurance Scheme; SD, standard deviation; SHS, senior high school; UQ, upper quartile.

**Table 4:** Sociodemographic characteristics of focus group participants in selected urban communities.

**Group participants (** **n** **)**	**Age (years)**	**Number of participants (** **n** **)**
FGD 1	40–56	5
Trader (5)		
FGD 2	41–67	5
Unemployed (1)		
Housewife (1)		
Caterer (1)		
Trader (2)		
FGD 3	40–51	4
Hairdresser (1)		
Housewife (1)		
Self-employed (1)		
Trader (1)		
FGD 4	42–52	8
Health officer (1)		
Administrator (2)		
Health inspector (5)		
FGD 5	40–58	8
E.H (1)		
Manager (1)		
Embossment officer (1)		
Secretary (2)		
Public servant (3)		
Total		30

**Table 5:** Attitudes and practices related to the use of mobile phones and SMS messages among Ghanaian women in selected urban communities.

**Characteristics**	**Community**	**Formal institutions**	**Total**	**p** ** value**
	**n** ** (%)**	**n** ** (%)**	**n** ** (%)**	
Received SMS messages				1.000
Yes	68 (98.6)	61 (100.0)	129 (99.2)	
No	1 (1.4)	0 (0.0)	1 (0.8)	
Normally read SMS messages				0.602
Yes	67 (98.5)	59 (96.7)	126 (97.7)	
No	1 (1.5)	2 (3.3)	3 (2.3)	
Ever received SMS messages on health-related topics				0.031
Yes	33 (47.8)	42 (68.9)	75 (57.7)	
No	30 (43.5)	18 (29.5)	48 (36.9)	
Cannot remember				
Willing to receive SMS messages on breast cancer screening test				0.046
Yes	69 (100.0)	57 (93.4)	126 (96.9)	
No	0 (0.0)	4 (6.6)	4 (3.1)	
Willing to go for breast cancer screening upon receipt of SMS messages				0.747
Yes	62 (89.9)	55 (90.1)	117 (90.0)	
No	3 (4.3)	4 (6.6)	7 (5.4)	
Do not know	4 (5.8)	2 (3.3)	6 (4.6)	
Preferred time of day to receive SMS messages on breast cancer				0.053
Morning	4 (5.8)	5 (8.2)	9 (6.9)	
Afternoon	4 (5.8)	6 (9.8)	10 (7.7)	
Evening	10 (14.5)	13 (21.3)	23 (17.7)	
Night	4 (5.8)	2 (3.3)	6 (4.6)	
Any time of the day	45 (65.2)	26 (42.6)	71 (54.6)	
Undecided	2 (2.9)	9 (14.8)	11 (8.5)	
Preferred duration of SMS messages broadcast				0.573
Less than 1 month	3 (4.3)	1 (1.6)	4 (3.1)	
1 month	12 (17.4)	12 (19.7)	24 (18.5)	
2 months	6 (8.7)	2 (3.3)	8 (6.2)	
More than 2 months	16 (23.2)	12 (19.7)	28 (21.5)	
Undecided	32 (46.4)	34 (55.7)	66 (50.8)	
Desired frequency of text messages per day				0.469
Once	27 (39.1)	28 (45.9)	55 (42.3)	
Twice	12 (17.4)	10 (16.4)	22 (16.9)	
Thrice	4 (5.8)	4 (6.6)	8 (6.2)	
More than thrice	2 (2.9)	5 (8.2)	7 (5.4)	
Undecided	24 (34.8)	14 (22.9)	38 (29.2)	

*Note:* %: column percentage.

**Table 6:** Views of Ghanaian women in selected urban communities on 35 SMS messages—findings from the SMS message pretesting (*n* = 3).

**Views of respondents**	**Frequency**
Received all SMS messages (yes)	3/3
Ever read the message (yes)	3/3
Clarity/ease of understanding message (yes)	3/3
Adequacy of text length/volume (yes)	3/3
Relevance of SMS messages increasing uptake of breast cancer screening (yes)	3/3
Willing to receive SMS reminders on breast cancer screening from the National Noncommunicable Disease Control Program (yes)	3/3
Time of day when text messages are read	
During working hours	2/3
After closing work	1/3
Timing of messaging was appropriate (yes)	2/3
SMS messages influenced my views on breast cancer screening (yes)	3/3

## Data Availability

All data supporting this manuscript are in the manuscript and supporting information files. All data transcripts coded and analyzed during this study are included in this article.

## Supporting Information

Supporting Information 1 File S1: COREQ checklist.

Supporting Information 2 File S2: STROBE checklist.

Supporting Information 3 File S3: survey respondents' suggestions for the types of breast cancer information to incorporate in SMS messages.

Supporting Information 4 File S4: proposed SMS messages developed during the stakeholder meeting.

Supporting Information 5 File S5: final SMS test messages developed for the main intervention study.
