# Supplementary material for: Development of a Culturally Appropriate Text Messaging Platform for Improving Breast Cancer Screening Uptake Among Ghanaian Women in Metropolitan Areas
Source: Int J Breast Cancer. 2024 Oct 24;2024:5587515. doi: 10.1155/2024/5587515 (PMC11527544; doi:10.1155/2024/5587515)
Supplement: Supporting Information 3 — File S3: survey respondents' suggestions for the types of breast cancer information to incorporate in SMS messages. [file 5587515.f3.docx]

**Supplementary file 3: Survey respondents' suggestions for the types of breast cancer information to incorporate in SMS messages**

| **Characteristics** | **Frequency** | **Percentage (%)** | |
| --- | --- | --- | --- |
| What Breast cancer is; | 8 | 6.15 |  |
| What Breast cancer is; Causes/Risk factors; | 16 | 12.31 |  |
| What Breast cancer is; Causes/Risk factors; Prevention | 6 | 4.62 |  |
| What Breast cancer is; Causes/Risk factors; Prevention; Treatment; | 4 | 3.08 |  |
| What Breast cancer is; Causes/Risk factors; Prevention; Treatment; Importance of Mammogram; | 1 | 0.77 |  |
| What Breast cancer is; Causes/Risk factors; Prevention; Treatment; Importance of Mammogram; Cost of Mammogram; | 1 | 0.77 |  |
| What Breast cancer is; Causes/Risk factors; Prevention; Importance of Mammogram; | 1 | 0.77 |  |
| What Breast cancer is; Causes/Risk factors; Prevention; Where to go for Mammogram; | 1 | 0.77 |  |
| What Breast cancer is; Causes/Risk factors; Prevention; Cost of Mammogram; | 2 | 1.54 |  |
| What Breast cancer is; Causes/Risk factors; Prevention; Other | 3 | 2.31 |  |
| What Breast cancer is; Causes/Risk factors; Treatment; | 1 | 0.77 |  |
| What Breast cancer is; Causes/Risk factors; Treatment; Importance of Mammogram; What to do if Mammogram is positive; | 1 | 0.77 |  |
| What Breast cancer is; Causes/Risk factors; Treatment; Where to go for Mammogram; | 2 | 1.54 |  |
| What Breast cancer is; Causes/Risk factors; Treatment; Other | 2 | 1.54 |  |
| What Breast cancer is; Causes/Risk factors; Importance of Mammogram; Where to go for Mammogram; | 1 | 0.77 |  |
| What Breast cancer is; Causes/Risk factors; Importance of Mammogram; Other | 1 | 0.77 |  |
| What Breast cancer is; Causes/Risk factors; Other | 4 | 3.08 |  |
| What Breast cancer is; Prevention; | 4 | 3.08 |  |
| What Breast cancer is; Importance of Mammogram; | 1 | 0.77 |  |
| Causes/Risk factors; | 13 | 10.0 |  |
| Causes/Risk factors; Prevention; | 7 | 5.38 |  |
| Causes/Risk factors; Prevention; Treatment; | 2 | 1.54 |  |
| Causes/Risk factors; Prevention; Treatment; Importance of Mammogram; Cost of Mammogram; | 1 | 0.77 |  |
| Causes/Risk factors; Prevention; Treatment; Importance of Mammogram; Cost of Mammogram; Other | 1 | 0.77 |  |
| Causes/Risk factors; Prevention; Treatment; Other | 1 | 0.77 |  |
| Causes/Risk factors; Prevention; Importance of Mammogram; | 2 | 1.54 |  |
| Causes/Risk factors; Prevention; Importance of Mammogram; Other | 1 | 0.77 |  |
| Causes/Risk factors; Prevention; Where to go for Mammogram; Cost of Mammogram; | 2 | 1.54 |  |
| Causes/Risk factors; Prevention; Where to go for Mammogram; Other | 1 | 0.77 |  |
| Causes/Risk factors; Prevention; Cost of Mammogram; Other | 2 | 1.54 |  |
| Causes/Risk factors Prevention; Other | 4 | 3.08 |  |
| Causes/Risk factors; Treatment; Where to go for Mammogram; Cost of Mammogram; Other | 1 | 0.77 |  |
| Causes/Risk factors; Treatment; Other | 1 | 0.77 |  |
| Causes/Risk factors; Where to go for Mammogram; Other | 1 | 0.77 |  |
| Causes/Risk factors; Cost of Mammogram; Other | 1 | 0.77 |  |
| Causes/Risk factors; Other | 8 | 6.15 |  |
| Prevention; | 2 | 1.54 |  |
| Prevention; Other | 2 | 1.54 |  |
| Treatment; | 1 | 0.77 |  |
| Importance of Mammogram; Other | 2 | 1.54 |  |
| Where to go for Mammogram; Cost of Mammogram; Other | 3 | 2.31 |  |
| Other | 11 | 8.46 |  |
| **Total** | **130** | **100.00** |  |
