# Supplementary material for: Development of a Culturally Appropriate Text Messaging Platform for Improving Breast Cancer Screening Uptake Among Ghanaian Women in Metropolitan Areas
Source: Int J Breast Cancer. 2024 Oct 24;2024:5587515. doi: 10.1155/2024/5587515 (PMC11527544; doi:10.1155/2024/5587515)
Supplement: Supporting Information 4 — File S4: proposed SMS messages developed during the stakeholder meeting. [file 5587515.f4.docx]

**Supplementary file 4: Proposed SMS messages developed during the stakeholder meeting**

| Goal:-  To increase the uptake of Screening among Ghanaian women using Text messages |
| --- |
| Communication objectives:-  1. Knowledge of Breast cancer  2. Knowledge of the importance of early detection  3. Allay Fear as a barrier  4. Encourage them to have time for their health  5. Accessibility to screening sites/cost |
| Objective 1: Knowledge of Breast cancer  1. Have you heard? Breast Cancer is the most common cancer in women in Ghana. Have your Breast checked!  2. About 2000 women get Breast cancer every year in Ghana.  3. Report any change in your Breast at a hospital.  4. If you noticed a lump in your Breast. Ask a Doctor. It may not be Cancer!  5. Don’t wait for pain in your Breast before you see a Doctor.  6. Breast cancer can be cured if detected and treated early.  7. Reporting late to the Doctor makes treatment difficult  8. Breast cancer is not a spiritual disease. It can be treated in a hospital  9. If you have a family member with Breast cancer, your risk may be increased. Have your Breast checked! |
| Objective 2: Knowledge of the importance of early detection  1. Do not let Breast cancer take you away from your loved ones. Have you checked your Breast yet?  2. Breast cancer should not kill you. You kill Breast cancer! Have your Breast checked!  3. Your breast can be saved if Breast cancer is detected early.  4. Do not let Breast cancer kill your dreams. Have your breast checked!  5. Ghana needs you alive. Have your Breast checked!  6. Early detection of Breast cancer reduces the cost of treatment. |
| Objective 3: Allay Fear as a barrier  1. Breast cancer screening is simple and not harmful! Have your Breast checked!  2. Breast cancer is curable when detected early. Have your Breast checked!  3. Breast cancer treatment does not kill/poisonous. Have your Breast checked!  4. 1 out of every 4 women who have their breasts checked regularly are saved from breast cancer/prevented from breast cancer deaths  5. 9 out of every 10 women who have early detection and treatment for breast cancer are still alive after 5 years  6. Having Breast cancer is not a death sentence. It can be treated. Have your Breast checked! |
| Objective 4: Encourage them to have time for their health  1. Your Health is your Wealth. Make time for your Health.  2. Have time for Breast cancer screening today. Save your life tomorrow  3. Spend time on your health. It is not time wasted.  4. Take time off your busy schedules and get screened for Breast cancer  5. A short time spent on Breast cancer screening saves a long stay at the hospital for treatment.  6. Have you had your breast checked? Make time for it today!  7. Breast cancer screening does not take long. You spend less time testing for Breast cancer than you spend at the hairdresser’s |
| Objective 5: Accessibility to screening sites/cost  1. Breast cancer screening is available in Ghana.  You can visit any of the following facilities:  i. Korle-Bu Teaching Hospital, Breast Clinic, Tuesdays, 10 am-3 pm.  ii. Trust Hospital, Osu  iii. Sunshine Center, Labadi  iv. Medical Imaging, Roman Ridge  v. Ridge Hospital, Accra  vi. C and J Center, Sakumono  vii. Diagnostic Center, East Legon  viii. Supreme Medical Center, Korle-Bu  ix. Sinel Specialist Hospital, Tema  2. The cost of breast cancer screening is far less than the cost of treatment  3. It is better to spend your pocket money today for screening than to spend your life savings on treatment tomorrow |
