# Supplementary material for: Development of a Culturally Appropriate Text Messaging Platform for Improving Breast Cancer Screening Uptake Among Ghanaian Women in Metropolitan Areas
Source: Int J Breast Cancer. 2024 Oct 24;2024:5587515. doi: 10.1155/2024/5587515 (PMC11527544; doi:10.1155/2024/5587515)
Supplement: Supporting Information 5 — File S5: final SMS test messages developed for the main intervention study. [file 5587515.f5.docx]

**Supplementary file 5: Final SMS test messages developed for the main intervention study**

| **CODE** | **MESSAGE** |
| --- | --- |
| Message BC1 | Have you heard? Breast Cancer is the most common cancer in women in Ghana. Have your Breast checked! |
| Message BC2 | About 2000 women get Breast cancer every year in Ghana. Have your Breast checked! |
| Message BC3 | Report any change in your Breast at a hospital as soon as possible. |
| Message BC4 | If you noticed a lump in your Breast. Ask a trained health provider. It may not be Cancer! |
| Message BC5 | Don’t wait for pain in your Breast before you see a trained health provider. |
| Message BC6 | Breast cancer can be cured if detected and treated early. Have your Breast checked! |
| Message BC7 | Reporting late to the trained health provider makes treatment difficult. Have your Breast checked as soon as possible! |
| Message BC8 | Breast cancer is not a spiritual disease. It can be treated in a hospital. Have your Breast checked! |
| Message BC9 | Breast cancer screening is available in Ghana.  You can visit any of the following facilities:  i. Korle-Bu Teaching Hospital, Breast Clinic, Tuesdays, 10 am-3 pm.  ii. Trust Hospital, Osu  iii. Sunshine Center, Labadi  iv. Medical Imaging, Roman Ridge  v. Ridge Hospital, Accra  vi. C and J Center, Sakumono  vii. Diagnostic Center, East Legon  viii. Supreme Medical Center, Korle-Bu  ix. Sinel Specialist Hospital, Tema |
| Message BC10 | If you have a family member with Breast cancer or a history of breast cancer, your risk may be increased. Have your Breast checked! |
| Message BC11 | Do not let Breast cancer take you away from your loved ones. Have you checked your Breast yet? |
| Message BC12 | Breast cancer should not kill you. You kill Breast cancer! Have your Breast checked! |
| Message BC13 | Your breast can be saved if Breast cancer is detected early. Have your Breast checked now! |
| Message BC14 | Do not let Breast cancer kill your dreams. Have your breast checked! |
| Message BC15 | Ghana needs you alive. Have your Breast checked as soon as possible! |
| Message BC16 | Early detection of Breast cancer reduces the cost of treatment. Have your Breast checked! |
| Message BC17 | Breast cancer screening is simple and not harmful! Have your Breast checked! |
| Message BC18 | Breast cancer screening is available in Ghana.  You can visit any of the following facilities:  i. Korle-Bu Teaching Hospital, Breast Clinic, Tuesdays, 10 am-3 pm.  ii. Trust Hospital, Osu  iii. Sunshine Center, Labadi  iv. Medical Imaging, Roman Ridge  v. Ridge Hospital, Accra  vi. C and J Center, Sakumono  vii. Diagnostic Center, East Legon  viii. Supreme Medical Center, Korle-Bu  ix. Sinel Specialist Hospital, Tema |
| Message BC19 | Breast cancer is curable when detected early. Have your Breast checked! |
| Message BC20 | Breast cancer treatment does not kill. It is not poisonous. Have your Breast checked! |
| Message BC21 | Many women who have their breasts checked regularly are saved from breast cancer and prevented from breast cancer death. |
| Message BC22 | Many women who have early detection and treatment for breast cancer are still alive after 5 years. |
| Message BC23 | Having Breast cancer is not a death sentence. It can be treated. Have your Breast checked! |
| Message BC24 | Your Health is your Wealth. Make time for your Health. Have your Breast checked! |
| Message BC25 | Have time for Breast cancer screening today. Save your life tomorrow. |
| Message BC26 | Spend time on your health. It is not time wasted. Have your Breast checked! |
| Message BC27 | Breast cancer screening is available in Ghana.  You can visit any of the following facilities:  i. Korle-Bu Teaching Hospital, Breast Clinic, Tuesdays, 10 am-3 pm.  ii. Trust Hospital, Osu  iii. Sunshine Center, Labadi  iv. Medical Imaging, Roman Ridge  v. Ridge Hospital, Accra  vi. C and J Center, Sakumono  vii. Diagnostic Center, East Legon  viii. Supreme Medical Center, Korle-Bu  ix. Sinel Specialist Hospital, Tema |
| Message BC28 | Take time off your busy schedules and get screened for Breast cancer. |
| Message BC29 | A short time spent on Breast cancer screening saves a long stay at the hospital for treatment. |
| Message BC30 | Have you had your breast checked? Make time for it today! |
| Message BC31 | Breast cancer screening does not take long. Have your Breast checked! |
| Message BC32 | Time spent on Breast cancer screening is time spent on your health. Have your Breast checked! |
| Message BC33 | The cost of breast cancer screening is far less than the cost of treatment. Have your Breast checked! |
| Message BC34 | It is better to spend your pocket money today for screening than to spend your life savings on treatment tomorrow. Have your Breast checked! |
| Message BC35 | Breast cancer screening is available in Ghana.  You can visit any of the following facilities:  i. Korle-Bu Teaching Hospital, Breast Clinic, Tuesdays, 10 am-3 pm.  ii. Trust Hospital, Osu  iii. Sunshine Center, Labadi  iv. Medical Imaging, Roman Ridge  v. Ridge Hospital, Accra  vi. C and J Center, Sakumono  vii. Diagnostic Center, East Legon  viii. Supreme Medical Center, Korle-Bu  ix. Sinel Specialist Hospital, Tema |
